# Nanobiosensors for Non-Amyloidbeta-Tau Biomarkers as Advanced Reporters of Alzheimer’s Disease

**DOI:** 10.3390/diagnostics10110913

**Published:** 2020-11-08

**Authors:** Le Minh Tu Phan, Thi Xoan Hoang, Thuy Anh Thu Vo, Jae Young Kim, Sang-Myung Lee, Won Woo Cho, Young Hyo Kim, Seong Hye Choi, Sungbo Cho

**Affiliations:** 1 Department of Electronic Engineering, Gachon University, Seongnam-si 13120, Gyeonggi-do, Korea; 2 School of Medicine and Pharmacy, The University of Danang, Danang 550000, Vietnam; 3 Department of Life Science, Gachon University, Seongnam 461-701, Gyeonggi-do, Korea; xoanht89@gmail.com (T.X.H.); vtathu0612@gmail.com (T.A.T.V.); jykim85@gachon.ac.kr (J.Y.K.); 4 Cantis Inc., Ansan-si 15588, Gyeonggi-do, Korea; smlee@cantis.co.kr (S.-M.L.); wwcho@cantis.co.kr (W.W.C.); 5 Department of Otorhinolaryngology-Head and Neck Surgery, School of Medicine, Inha University, Incheon 22332, Korea; inhaorl@inha.ac.kr; 6 Department of Neurology, School of Medicine, Inha University, Incheon 22332, Korea; seonghye@inha.ac.kr; 7 Department of Health Sciences and Technology, GAIHST, Gachon University, Incheon 21999, Korea

**Keywords:** Alzheimer’s disease, non-Aβ-Tau biomarkers, advanced reporters, nanomaterial, biosensor

## Abstract

Emerging nanomaterials providing benefits in sensitivity, specificity and cost-effectiveness are being widely investigated for biosensors in the application of Alzheimer’s disease (AD) diagnosis. Core biomarkers amyloid-beta (Aβ) and Tau have been considered as key neuropathological hallmarks of AD. However, they did not sufficiently reflect clinical severity and therapeutic response, proving the difficulty of the Aβ- and Tau-targeting therapies in clinical trials. In recent years, there has still been a shortage of sensors for non-Aβ-Tau pathophysiological biomarkers that serve as advanced reporters for the early diagnosis of AD, predict AD progression, and monitor the treatment response. Nanomaterial-based sensors measuring multiple non-Aβ-Tau biomarkers could improve the capacity of AD progression characterization and supervised treatment, facilitating the comprehensive management of AD. This is the first review to principally represent current nanobiosensors for non-Aβ-Tau biomarker and that strategically deliberates future perspectives on the merit of non-Aβ-Tau biomarkers, in combination with Aβ and Tau, for the accurate diagnosis and prognosis of AD.

## 1. Introduction

Alzheimer’s disease (AD) is characterized as a progressive neurodegenerative disorder that causes memory deficits and cognitive impairment. Pathologically, AD is associated with the formation of senile plaques and neurofibrillary tangles in the brain by the accumulations of aggregated amyloid-β (Aβ) and Tau proteins, which are considered as central hallmarks in AD [1,2,3]. The sustained inflammatory response in the AD patient brain emerged as third core pathology which contributes to the onset and progression of AD, suggesting that it is a feasible target for therapeutic intervention [4]. Along with neurodegeneration evaluation, the simultaneous monitoring of Aβ and Tau biomarkers have efficaciously certified in the early diagnosis of AD [5]. The measurement of two core biomarkers has been utilized for the assessment of AD in both the preclinical stage and treatment effectiveness on Aβ and Tau pathologies. However, the accurate prediction of disease progression and therapeutic response does not consistently rely on the fluctuation of Aβ and Tau levels, limiting the beneficial efficacy of Aβ and Tau in AD management. The AD duration and severity do not correlate well with the concentrations of Aβ and Tau in cerebrospinal fluid (CSF) [6] due to the early saturation of Aβ accumulation in the brain before clinical sign appearance [7,8] and the change of Tau levels during the developmental neurodegeneration [9,10]. Hence, diagnostic tools for non-Aβ-Tau biomarkers as advanced reporters in cooperation with Aβ and Tau detection are essential to enable the early diagnosis, accurate observation of progression and therapeutic effects of AD.

Nanomaterial-based biosensors have emerged as modern detection technologies for AD diagnosis due to their advantages such as sensitivity and selectivity enhancement, simplicity, and cost-effectiveness. However, Aβ and Tau protein are by far the most attractive to scientists in nanobiosensor development due to their representative characteristics of AD [11,12,13]. Meanwhile, non-Aβ-Tau biomarkers have been considered less attractive to investigate nanomaterial-based sensors for monitoring AD progression and therapeutic effects. This review primarily focuses on the nanobiosensors of non-Aβ-Tau biomarkers for the potential improvement of the diagnosis and monitoring of AD progression and therapeutic effect. The biosensors are classified into two critical features known as the optical and electrochemical biosensor that provide a comprehensive detection limit comparison of non-Aβ-Tau targets. Additionally, the innovative approaches for the measurement of non-Aβ-Tau biomarkers in combination with Aβ and Tau have been strategically discussed to identify the standardized diagnostic techniques towards the optimal controlling effort of AD.

## 2. Importance of Non-Aβ-Tau Biomarkers in Monitoring Alzheimer’s Disease

Currently, AD diagnoses are having to face enormous challenges in which the clinical symptoms occur decades after accumulating neuropathological modifications [14]. It is well known that extracellular Aβ deposition and the intracellular hyperphosphorylation of Tau proteins are general considerations for AD’s diagnostic biomarkers and various hypotheses have been put forth to shed light on the pathogenesis from multi-omics studies [14,15]. Aβ monomers generally consist of 36–43 amino acids; however, the Aβ42/40 ratios in CSF, usually measured by immunoassays or Aβ positron emission tomography (PET) imaging are most broadly evaluated that reflect Aβ aggregation and subsequent senile plaques formation [15,16,17]. In parallel with amyloidosis, Tau, a microtubule-binding protein phosphorylated and accumulated into neurofibrillary tangles (NFT), is reflected as a second biomarker for AD [17]. In terms of AD prediction, total Tau (T-tau), as well as Tau phosphorylated at threonine 181 (P-tau), are the core CSF predictors [18]. In normal physiological conditions, Aβ functions to regulate learning and memory, neurogenesis, angiogenesis and repair leaks in the blood–brain barrier (BBB), etc., while Tau protein also holds several nerve-related essential roles such as myelination, axonal transport, neuronal excitability, microtubule dynamics, so on [19]. Nevertheless, various *in vitro* studies revealed that upon the challenge of synthetic Aβ42, the observable results in the human induced pluripotent stem cell iPSC-derived neuron demonstrated several neuronal deficits such as neuronal death, ER stress or synaptotoxicity [20]. Furthermore, the high Aβ42/40 ratio can robustly induce Tau hyperphosphorylation and perhaps neurodegeneration [20]. In turn, Mclnnes’s group indicated that the interaction between Tau and synaptogyrin-3 lessened synaptic neurotransmitter release, as well as attenuated protein translation and nuclear transcription, consequently associated with neuronal dysfunction and cognitive decline [21]. From these reasons, Aβ and Tau species are the main targets of numerous studies to develop biosensors that allow the detection in both invasive samples such as CSF, plasma [22,23,24,25] and non-invasive samples such as saliva and urine [26,27].

**Figure 1:**
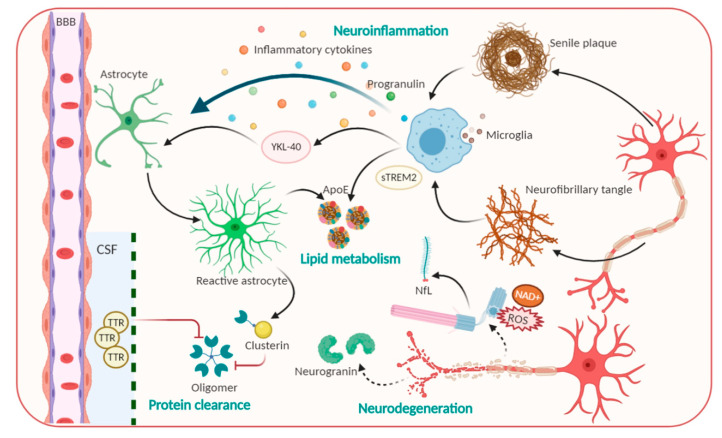
Pathophysiological processes including Amyloid beta, Tau and candidate non-Aβ-Tau biomarkers for Alzheimer’s disease.

Based on conventional understanding about AD pathology, numerous laboratory studies and clinical trials made intensive attempts to disrupt the refractory of AD via Aβ and Tau targeting [16]. Many studies are under different phases of evaluation; unfortunately, almost completed ones have been comprehensively futile because of facing primary cognitive outcomes, especially in phase III trial [16,28]. To further investigate an efficacious therapeutic target, remaining pathological alterations in the brain were considered, including inflammation, neurodegeneration, lipid metabolism, synaptic dysfunction, protein clearance, and mitochondrial dysfunction [17,29,30,31,32,33]. These modifications directly regulate preclinical AD toward persistent and multifaceted AD dementia [14]. Therefore, molecules associated with the multifaceted nature of AD pathophysiological progression have been considered as novel biomarkers in AD (Figure 1).

**Neurodegeneration-related biomarkers: New promising candidates for AD diagnosis**. Neurodegeneration is not only inevitable but also exacerbated in AD progression [33], as various neuronal and synaptic-related proteins which are most associated with brain development have been suggested to be involved in the first step of AD progression, and their function precedes neuronal loss, thus allowing them to be considered as CSF biomarkers for AD. Typically, visinin-like protein 1 (VLP-1) can seep out from dented neurons and act as a vital calcium sensor protein. VLP-1 was shown to be significantly increased in AD, suggesting it as a useful biomarker that correlates with the degree of dementia. Currently, combined analyses of Aβ, P-tau, and VLP-1 have been performed and were reported to increase the accuracy of AD diagnosis [34,35]. Furthermore, growth-associated protein, which is another synaptic protein involved in the regulation of axonal outgrowth, synaptic plasticity, and learning and memory functions, was found to be present at higher levels in CSF [34,36]. Particularly, neurofilament light (NfL) polypeptide, an axonal cytoskeleton composition, is leaked from axonal injury into brain interstitial fluid, then tracked into CSF and blood [16,37]. Previous studies reported that NfL concentration is elevated approximately 16 years before the judgment of disease onset. Measuring the NfL level can be taken place in CSF and blood samples for hypometabolism and neurodegeneration, especially with changing cognitive scores. For these reasons, NfL elevated rates express as a great feature for the cost-effective and non-invasive diagnostic measurement of a broad range of neurodegeneration diseases, as well as clinical progression in pre-symptomatic of AD [16,38].

**Neuroinflammation and phagocytosis of an innate immune system: Potential therapeutic targets**. The propagation of phagocytosis and the inflammatory process, which are involved in the initiation and exacerbation of AD, are among the most attractive events for AD physiological behavior identification [39,40]. Indeed, microglia—brain resident macrophages—are responsible for microenvironmental surveillance, the clearance of debris and pathogens, and sustaining the secretion of proinflammatory mediators [39]. Additionally, conclusive evidence demonstrated that inflamed molecules, such as those in iNOS production, tend to speed up Aβ aggregation and senile plaques formation, ultimately leading to a precarious vicious cycle [39,41]. Of note, a triggering receptor expressed on myeloid cells 2 (TREM2), which is highly expressed in microglia, modulates plaque-surrounding microglial activities including survival, proliferation, cytokine release as well as biosynthetic metabolism [42]. Nevertheless, compelling evidence has revealed that the levels of the ectodomain of TREM2, which was proteolytic cleaved and liberated to generate extracellular soluble TREM2 (sTREM2), were elevated in the CSF in AD stage-dependent milieu [42,43]. sTREM2 not only recapitulated full-length TREM2-like functions but also contributes to recruiting microglia to the plaques. Significantly, Ewers group’s outcomes denoted that higher CSF sTREM2 levels are responsible for less cognitive decline in hippocampal volume [42,43]. Accordingly, higher CSF sTREM2 concentration may act as a biomarker representing the amelioration of pathological progression at the AD’s symptomatic stage [43]. Besides microglia, another star-shaped glial cell—astrocytes—also play essential roles in Aβ phagocytosis and degradation, strengthening trophic nerves as well as generating a safety barrier between Aβ accumulation and neurons. However, a result reported that upon the chronic stress, astrocytes overexpress β-secretase (BACE1), which induce Aβ overproduction [44]. β2-microglobulin, intercellular adhesion molecule 1 (ICAM1), progranulin and chitinase-3-like protein 1 (CHI3L1/YKL-40) also participate in neuroinflammation, thus, affecting AD pathology [33]. YKL-40 was expressed in activated astrocytes and microglia [30] whose level is associated with an enhanced early AD continuum and exacerbated neuroinflammation; thus, it exerts the features of a promising biomarker for AD [14]. Furthermore, molecules related to the uptake and degradation of unfolded Aβ and hyperphosphorylated Tau, have received much more interest as potential biomarkers. Typically, transthyretin (TTR) or clusterin, are those that are elevated in CSF, and act as a molecular chaperon that can directly bind to the Aβ molecule to prevent Aβ accumulation and the resultant attenuated Aβ-associated cellular toxicity [14,33]. Hence, these factors perform protective activities against the excessive Aβ load, thereby serving as a potential candidate for stage and state AD diagnosis.

**Lipid metabolism biomarker**. Lipid metabolites are highly associated with AD progression; thus, they have been investigated as promising disease biomarkers [32]. The first biomarker that markedly increases the risk for developing AD is ApoE, the molecule that is involved in the normal catabolism of triglyceride-rich lipoproteins and exhibits immunoreactivity in Aβ deposits and NFTs. ApoE is a glycoprotein that is highly expressed in the brain. This glycoprotein contains 299 amino acids and is classified into three common isoforms in humans that differ in their structures [45,46]. ApoE regulates the isoform-dependent removal of Aβ, via Aβ lipoprotein complexes endocytosis, by influencing proteolytic degradation of Aβ and facilitating its transport across BBB [14]. In addition, ApoE has been shown to influence microglial activation states and cellular responses in a TREM2-dependent way; especially ApoE-knockdown in mice blocks microglial phagocytic function to Aβ [47]. Numerous studies imply that ApoE4 harmfully accelerates Aβ aggregation by interacting with Aβ to promote Aβ aggregation and to stabilize Aβ oligomers. On the other hand, other pieces of evidence showed that ApoE2 exerts a protective function in AD [48]. Therefore, the quantification of isoform-dependent ApoE levels is promising as a CSF diagnostic biomarker.

## 3. Optical Sensors for Detection of Non-Aβ-Tau Biomarkers

Due to distinct advantages including high specificity, sensitivity, and cost-effectiveness, the optical biosensors for non-Aβ-Tau biomarkers are able to conduct the label-free and real-time detection of targets. Analyte concentrations are consistently equivalent to signals from the optical transducer system that exhibits the optical intensities after target biorecognition [49]. Optical biosensors are nanomaterially categorized as fluorescent biosensors, colorimetric biosensors, localized surface plasmon resonance (LSPR) sensors are mainly developed to quantify non-Aβ-Tau biomarkers for the monitoring of AD. The recent nanomaterial-based optical biosensor advances with regard to other biofluid markers of AD are summarized in Table 1 to represent the sensing performance for these biomarkers.

**Figure 2:**
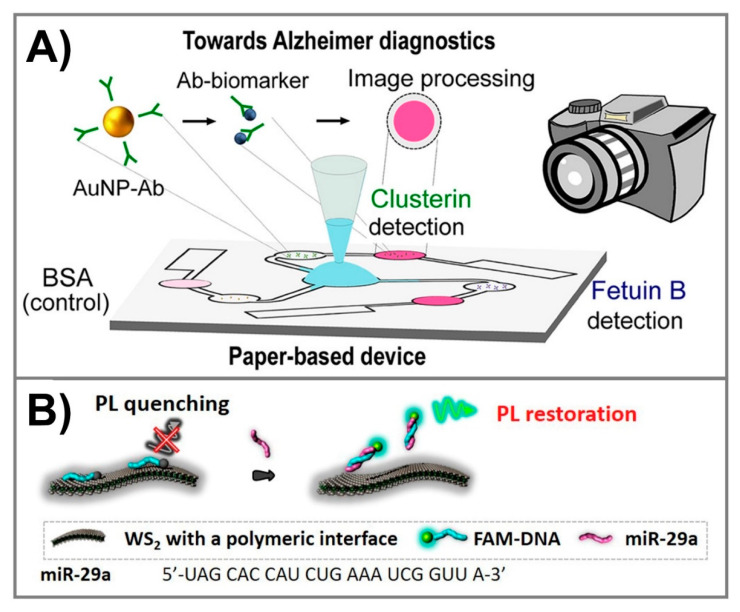
(**A**) Colorimetric lateral flow paper device using AuNPs for the detection of clusterin and fetuin B biomarkers of Alzheimer’s disease (AD). Adapted with permission from Ref. [51]. (**B**) Schematic depiction for the fluorescent detection of the miR-29a AD biomarker on WS2 nanosheets with polymeric interfaces. Adapted with permission from Ref. [59].

Dual-readout (colorimetric and fluorometric) assays for the detection of acetylcholinesterase (AChE) in CSF were developed using Rhodamine B and gold nanoparticles (AuNPs). Rhodamine B, a strongly fluorescent chemical, was adsorbed onto surfaces of AuNPs and facilitated the fluorescence quenching of rhodamine B. When both acetylthiocholine and AChE were added to the AuNPs@rhodamine B solution, AChE hydrolyzed acetylthiocholine to generate thiocholine. Due to the strong binding affinity of thiocholine onto surfaces of AuNPs via an the Au–S bond, thiocholine could replace the position of rhodamine B molecules, thus resulting in the detachment of rhodamine B molecules from AuNPs to recover rhodamine B fluorescence and facilitate the aggregation of AuNPs. This assay could measure AChE within 20 min at limit of detection (LOD) of 1 mU/mL according to AuNP aggregation-based colorimetric detection. Notably, this method showed an improvement for rhodamine B monitoring with LOD of 0.1 mU/mL, implying its sensitivity for the diagnosis of AD [50]. Fetuin B and clusterin have also been reported to be related to AD. A paper-based lateral flow immunoassay (LFA) for the simultaneous determination of fetuin B and clusterin was developed using AuNPs (Figure 2A). The biofluid containing the biomarkers flows laterally toward the selective antibody, thus permitting AuNP@antibody accumulation on the test zone and leading to a color change from white to pink. The specific antibodies with high affinity to fetuin B and clusterin were firstly immobilized onto the surface of AuNPs, respectively, making the effective colorimetric probes to detect the appearance of these antigens. Competitive and sandwich immunoassays were applied for the quantification of fetuin B and clusterin within 15 min at detection limits of 0.24 nM and 0.12 nM, respectively, thus providing a rapid and sensitive paper-based device that could be used for the detection of multiple AD biomarkers to achieve a more effective AD diagnosis [51]. Fluorescent biosensors were also applied for the detection of AD biomarkers. For example, graphene oxide (GO) and up-conversion nanoparticles (UCNPs) were used for mRNA-related oligonucleotides [57], CdSe@ZnS fluorescent quantum dots (QDs) for apolipoprotein E [55], and WS2 nanosheets and fluorescein (FAM) for miR-29a [59]. Due to the potential of transition metal dichalcogenides to act as fluorescence quenchers for the rapid detection of DNAs and miRNAs in the Forster resonance energy transfer-based assays, WS2 nanosheets were generated for the fluorescence-based detection of miR-29a, the microRNAs corresponding to the formation of toxic Aβ peptides (Figure 2B). The WS2 nanosheets functionalized by trimethylammonium-modified dextran exhibited the rapid adsorption of the fluorescein-labeled DNA probe (FAM-DNA), thus leading to the effective photoluminescence (PL) quenching of FAM. Following the addition of miR-29a, hybridization between miR-29a and the complementary FAM-DNA probe resulted in the desorption of FAM-DNA from WS2 nanosheets to recover the fluorescence. The WS2 based sensor could detect miR-29a at LOD of 745 pM against non-complementary and the single base-mismatched RNA in human serum [59]. The optical biosensors for non-Aβ-Tau biomarkers can be used in conjunction with biosensors for Aβ and Tau protein to provide a sensitive diagnostic tool for the accurate diagnosis and observation of progression or the therapeutic effects of AD.

## 4. Electrochemical Sensors for Detection of Non-Aβ-Tau Biomarkers

Electrochemical sensors are widely utilized for the measurement of biotargets due to high sensitivity, equipment minimization and portability [62]. To improve electrochemical sensing performance, various functional nanomaterials have been fabricated to be employed in electrochemical biosensors [63,64,65]. Related to AD disease diagnosis, non-Aβ-Tau biomarkers including ApoE protein [66], ApoE-encoding gene [67,68], AD-related DNA [69,70], microRNA [71], α-1 antitrypsin [72], β-secretase [73], and immunoglobulin [74], have been successfully detected electrochemically using various nanomaterial-based sensing strategies. Different nanostructure-modified electrochemical biosensors were summarized in Table 2 to compare the limit of detection and emphasize their advantages in the early diagnosis of AD as well as monitor therapeutic response through the quantification of low-abundance biomarkers.

**Figure 3:**
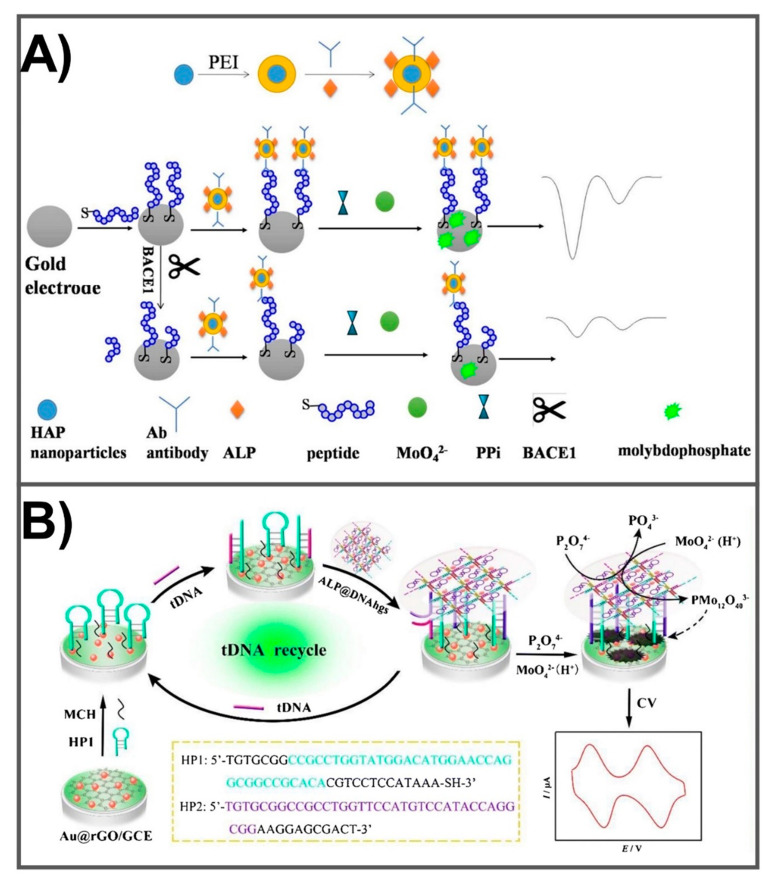
(**A**) Fabrication of electrochemical sensor to detect BACE1 by employing the combination of hydroxyapatite (HAP) nanoparticle and alkaline phosphatase (ALP) as dual signal amplification. Adapted with permission from Ref. [73]. (**B**) The preparation of ALP-wrapped DNA hydrogel-based electrochemical biosensing for AD-related DNA markers. Adapted with permission from Ref. [69].

A rapid, easy, and sensitive assay has been introduced by adopting the enzymatic cleavage activity of the restriction enzyme HhaI and the signal amplification of Fc-capped AuNPs/streptavidin for the discrimination and quantification of the ApoE4 gene. This sensing method allowed for the detection of ApoE4 at levels as low as 0.1 pM [67]. In another study, the functionalization of screen-printed carbon electrodes with Iridium oxide nanoparticles (IrO_2_ NPs) resulted in a sensing system that exhibited high performance for the detection of ApoE protein at LOD of 68 ng/mL [66], which is much lower than the ApoE level found in the CSF of AD model (9.09 µg/mL) [75]. The αApoE antibody was immobilized onto an IrO_2_ NP surface (αApoE- IrO_2_ NP). Another complex included an αApoE antibody-modified magnetic bead (αApoE-MB) was prepared. Using a sandwich immunoassay principle, the conjugate of αApoE-IrO_2_ NP recognized the αApoE protein and formed a magnetosandwich complex (αApoE-IrO_2_ NP/αApoE/αApoE-MB). The electrochemical detection of αApoE in this magnetosandwich assay was determined using a water oxidation reaction. This novel detection method possessing a rapid, simple, and highly sensitive performance could be extended for use in the quantification of other AD biomarkers in a biological context. The sequential synthesis of Aβ is dependent upon the activity of protease β-site amyloid precursor protein cleaving enzyme (BACE1), the enzyme that catalyzes the first step in Aβ generation. A dual-signal amplification-based electrochemical sensing method was developed (BACE1 analysis) using hydroxyapatite nanoparticles (Figure 3A). For sensor fabrication, hydroxyapatite (HAP), a redox-generating nanoparticle was utilized as a probe. Upon the reaction between HAP probe and molybdate (MoO4-), an electrochemical current is generated. For dual signal amplification, alkaline phosphatase (ALP) and an antibody against Aβ were integrated onto the surface of the HAP nanoparticle. A gold electrode surface was modified by integrating peptides which can be cleaved by active BACE1. In the presence of BACE1, the peptides were cleaved to release the peptide fragment that in turn binds to Aβ antibody, leading to a decrease in HAP-ALP binding sites, resulting in a current intensity which is proportional to the concentration of BACE1. The enzymatic activity of BACE1 was detected at a concentration range from 0.25 to 100 U/mL with a detection limit down to 0.1 U/mL after a 60 min incubation time [73]. This dual-signal amplification strategy shows promise for use in the detection of other peptidases in a wide range of applications. Another sensitive strategy was developed for the detection of AD-related target DNA (tDNA) [69]. This fabrication method was based on alkaline phosphatase-packaged DNA hydrogels (ALP@DNAhg) that functioned to initiate tDNA recycling signal amplification. PMo_12_O_40_^3−^ was used as a redox mediator, immobilized onto an Au@rGO surface to promote electron transfer (Figure 3B). By exploiting these advantageous properties, an accurate, highly sensitive biosensor was developed that exhibited a linear range of detection from 1.0 × 10^−2^ to 1.0 × 10^4^ pM and LOD of 3.4 × 10^−3^ pM. Furthermore, immunoglobulins and autoantibodies have been identified as new biomarkers of AD [76,77]; however, the development of sensors for these molecules as biomarkers for AD has received less investigation. Typically, plasma immunoglobulin (Ig) was detected using an electrochemical platform by integrating polyclonal rabbit anti-human Ig as a receptor element on the gold electrode surface [74]. Upon exposure to plasma solutions, the interaction between target Ig and receptor leads to the change in surface properties which can be detected by electrochemical impedance spectroscopy and cyclic voltammetry. This sensor allowed the detection of IgG within 15 min with LOD down to pg/mL which represents plasma content concentrations [78]. With high sensitivity and applicability, the development of other sensing systems for the detection of CSF immunoglobulin and autoantibodies exhibits promising potential for AD diagnosis. Altogether, AD multi-marker measurements performed simultaneously in a single electrochemical detection system could significantly provide the early diagnosis and monitoring of the treatment therapy of AD.

## 5. Conclusions and Future Perspectives

Since Alzheimer’s disease is considered to be among the most prevalent neurodegenerative diseases, it is tremendously required to develop accurate techniques for the diagnosis and monitoring of disease progression in the early stages of AD. Practical monitoring approaches for AD have been mainly developed based on Aβ and Tau detection due to their most diagnostic value in the early stages of AD. Nevertheless, recent evidence has revealed that the hallmarks of AD neuropathology are more complicated than it appeared to be. A number of the inconsistent diagnostic results based on Aβ and Tau [79,80,81] has indicated that the attention on these core markers only is insufficient to predict the disease progression and monitor the treatment response. Besides Aβ and Tau pathological pathways, the alterations inside the brain also contribute to disease progression, including inflammation, neurodegeneration, and lipid metabolism. Therefore, it is critical to broaden the focus on biomarkers from others’ AD pathway, complementarily with Aβ and Tau, to develop biosensing tools for the accurate diagnosis and therapeutic response monitoring of AD.

**Figure 4:**
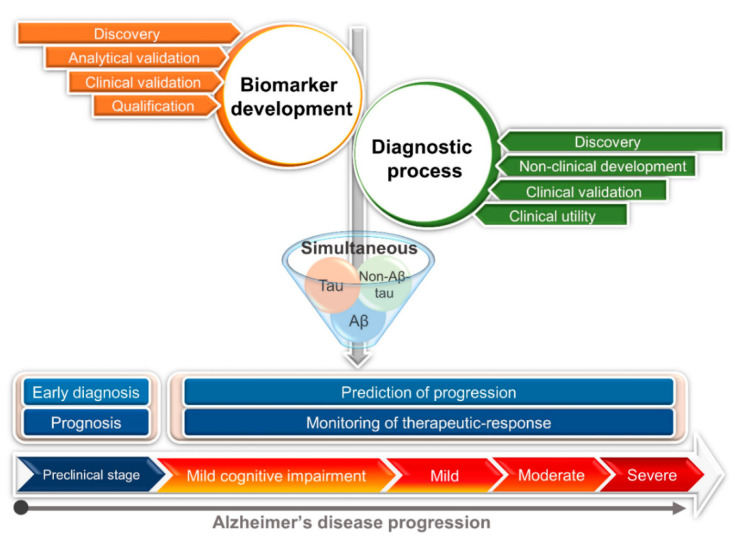
Future perspective for monitoring of AD at different clinical stages using nanobiosensors via the simultaneous measurement of Aβ, Tau and non-Aβ-Tau biomarkers.

Nanobiosensors targeting Aβ and Tau with high sensitivity have been successfully developed for the early diagnosis of AD before the manifestation of clinical symptoms. However, that is not always reliable enough for monitoring disease progression and treatment response at some particular clinical circumstances. In recent years, non-Aβ-Tau pathophysiological biomarkers such as apolipoprotein E, progranulin, visinin-like protein 1, sTREM2 have been identified to be promising reporters for diagnosis of AD progression, where the utility of Aβ and Tau may be inaccurate to give the correct conclusion. Additionally, the tracking of a single biomarker is often inadequate to specify the clinical status and therapeutic response of AD. To address these concerns, the redirection of diagnosis strategies towards the simultaneous monitoring of non-Aβ-Tau biomarkers in cooperation with Aβ and Tau will be necessary for the remarkable enhancement of diagnostic accuracy and treatment efficacy. Nanomaterial-based sensors for AD biomarkers have contributed to AD diagnostic impacts with high efficiency. In recent years, only several nanobiosensors for the detection of non-Aβ-Tau biomarkers have been developed to significantly improve the diagnostic validity. Therefore, the nanobiosensor systems for these new emerging biomarkers should receive more attention to monitor the AD stages and treatment response. The future perspectives of AD management through biomarker development and diagnostic process using nanobiosensors at different clinical stages are strategically deliberated in Figure 4. The new valuable biomarkers of AD are being explored and qualified to achieve more diagnosis efficacy. In parallel, the candidate nanobiosensors investigated for these new biomarkers should be discovered and utilized in monitoring of AD after clinical trials. The ideal sensing technology to detect multiple biomarkers (combination of Aβ, Tau and non-Aβ-Tau biomarkers) suggests more valuable diagnostic tools for AD monitoring. Along with the successful development of nanobiosensors for Aβ and Tau [12,13], the advanced nanobiosensors for measuring the appearance of AD complex biomarkers (Aβ, Tau and non-Aβ-Tau) play an important role to efficiently track the clinical stages of AD and monitoring the treatment response. These commercialized multivariate approaches would provide the individual diagnosis of AD for the improvement of the efficacy of AD management. Point-of-care sensors targeting these biomarkers should be under intensive attention and become available in a not-so-distant future, supporting their effort in the management and treatment of AD.

In conclusion, this review has offered the current trend in the development of nanobiosensors for non-Aβ-Tau advanced reporters. Nanomaterial-based optical and electrochemical sensors for the detection of emerging non-Aβ-Tau biomarkers exhibit the strong potential towards early diagnosis and treatment response monitoring. The ideal incorporation of these biomarkers with Aβ and Tau proteins provides superior diagnostic value for the accurate diagnosis at the precise stage of the disease. Furthermore, nanomaterial-based biosensors of these non-Aβ-Tau biomarkers with high sensitivity serve as desirable techniques to monitor the therapeutic response of AD. The nanobiosensors for the simultaneous detection of the complex of AD biomarkers (Aβ, Tau, and non-Aβ-Tau) will be beneficial to track disease progression and treatment response accurately, contributing to the optimal controlling efforts of AD.

## Figures and Tables

**Table 1:** Summary of the nanomaterial-based optical sensors of non-Aβ-Tau biomarkers.

Principle	Biomaterial	Nanomaterial	Target	Biological Sample	Time Response	LOD	Ref
Colorimetric	-	AuNPs	AChE	CSF	20 min	1 mU/mL	[50]
Fluorescent	-	AuNPs	AChE	CSF	20 min	0.1 mU/mL	[50]
LFA	Antibody	AuNPs	Clusterin	Plasma	15 min	0.12 nM	[51]
LFA	Antibody	AuNPs	Fetuin B	Plasma	15 min	0.24 nM	[51]
MRI	Antibody	MNPs	Ferritin	Mouse brain	360 min	-	[52]
LSPR	ssDNA	AuNPs	ApoE gene	-	120 min	512 nM	[53]
LSPR	Aβ40, Aβ42	AuNPs	ApoE4	CSF	Overnight	1.5 pM	[54]
Fluorescent	Antibody	CdSe@ZnS QDs	ApoE	Serum	210 min	62 pg/mL	[55]
SPR	Antibody, aptamer	-	α-1 Antitrypsin	Serum	60 min	10 fM	[56]
NIR fluorescent	ssDNA	NaYF4:Yb, Er UCNPs, GO	BACE-1 mRNA	Serum	60 min	500 fM	[57]
Colorimetric	ssDNA	AuNPs	miR-137	Plasma	120 min	0.25 nM	[58]
Fluorescent	DNA	WS2 nanosheets	miR-29a	Serum	100 min	745 pM	[59]
Fluorescent imaging	DNA	QDs	mRNA	Plasma	-	-	[60]
Fluorescent	DNA	-	DNA	-	60 min	200 pM	[61]

**Table 2:** Summary of the nanomaterial-based electrochemical sensors of non-Aβ-Tau biomarkers.

Platform	Biomaterial	Nanomaterial	Target	Biological Sample	Time Response	LOD	Ref
SPE	Antibody	IrO2 NPs	ApoE	Plasma	45 min	68 ng/mL	[66]
Gold electrode	DNA	-	ApoE4 gene	Serum	360 min	0.1 pM	[67]
GCE	DNA	GSHs	ApoE gene	-	60 min	10 fM	[68]
GCE	DNA	Au@rGO	tDNA	Serum	180 min	3.4 fM	[69]
DEP	DNA	GO	hpDNA	-	30 min	6.6 pM	[70]
SPE	DNA	GO AuNWs	miR-137	Serum	135 min	1.7 fM	[71]
SPE	Aptamer Antibody	PTCA-CNTs	α−1 antitrypsin	Serum	120 min	0.01 pM	[72]
Gold electrode	Peptide	-	β-secretase	Serum	60 min	0.1 U/mL	[73]
Gold electrode	Antibody	-	Immunoglobulin	Plasma	15 min	-	[74]
